# Identification of SPHK1 as a therapeutic target and marker of poor prognosis in cholangiocarcinoma

**DOI:** 10.18632/oncotarget.4335

**Published:** 2015-06-02

**Authors:** Ming-Huang Chen, Chueh-Chuan Yen, Chi-Tung Cheng, Ren-Chin Wu, Shih-Chiang Huang, Chung-Shan Yu, Yi-Hsiu Chung, Chun-Yu Liu, Peter Mu-Hsin Chang, Yee Chao, Ming-Han Chen, Yu-Fen Chen, Kun-Chun Chiang, Ta-Sen Yeh, Tzu Chi Chen, Chi-Ying F. Huang, Chun-Nan Yeh

**Affiliations:** ^1^ Institute of Clinical Medicine, National Yang-Ming University, Taipei, Taiwan; ^2^ Division of Hematology and Oncology, Department of Medicine, Taipei Veterans General Hospital, Taipei, Taiwan; ^3^ Department of Surgery, Chang Gung Memorial Hospital, Chang Gung University, Taoyuan, Taiwan; ^4^ Department of Pathology, Chang Gung Memorial Hospital, Chang Gung University, Taoyuan, Taiwan; ^5^ Department of Biomedical Engineering and Environmental Sciences, National Tsing-Hua University, Hsinchu, Taiwan; ^6^ Center for Advanced Molecular Imaging and Translation, Chang Gung Memorial Hospital, Taoyuan, Taiwan; ^7^ Department of Oncology, Taipei Veterans General Hospital, Taipei, Taiwan; ^8^ Department of General Surgery, Chang Gung Memorial Hospital, Keelung, Taiwan; ^9^ Institute of Clinical Medicine and Institute of Biopharmaceutical Sciences National Yang-Ming University, Taipei, Taiwan

**Keywords:** cholangiocarcinoma, sphingosine kinase 1, sphingosine-1-phosphate, SK1-I, sphingosine-1-phosphate receptor 2

## Abstract

Cholangiocarcinoma (CCA) is characterized by a uniquely aggressive behavior and lack of effective targeted therapies. After analyzing the gene expression profiles of seven paired intrahepatic CCA microarrays, a novel sphingosine kinase 1 (SPHK1)/sphingosine-1-phosphate (S1P) pathway and a novel target gene, SPHK1, were identified. We hypothesized that therapeutic targeting of this pathway can be used to kill intrahepatic cholangiocarcinoma (CCA) cells. High levels of SPHK1 protein expression, which was evaluated by immunohistochemical staining of samples from 96 patients with intrahepatic CCA, correlated with poor overall survival. The SPHK1 inhibitor SK1-I demonstrated potent antiproliferative activity *in vitro* and *in vivo*. SK1-I modulated the balance of ceramide-sphinogosine-S1P and induced CCA apoptosis. Furthermore, SK1-I combined with JTE013, an antagonist of the predominant S1P receptor S1PR2, inhibited the AKT and ERK signaling pathways in CCA cells. Our preclinical data suggest SPHK1/S1P pathway targeting may be an effective treatment option for patients with CCA.

## INTRODUCTION

Cholangiocarcinoma (CCA) is a relatively rare hepatobiliary cancer; however, the incidence and mortality of CCA are increasing worldwide [1-3]. Intrahepatic CCA is the second most common liver cancer, accounting for 10%–15% of all primary liver malignancies [4]. Surgical resection is the only potentially curative therapy for intrahepatic CCA patients; however, most cases are advanced at diagnosis, with poor liver function for which only palliative chemotherapy is available [4, 5].

The development of targeted therapies in cancer can be guided by the identification of tumor-associated pathways. Several pathways are deregulated in intrahepatic CCA, including IL-6/STAT3 signaling and growth factors such as EGF, HGF/MET, and VEGF, and the KRAS/MAPK and PI3K/AKT pathways [6-8]. Although these pathways contain several potential targets and molecular-targeted therapies have been assessed in clinical trials, all results have been negative and there remains no effective therapy for refractory CCA [8-9].

We used microarray analysis to identify other targetable pathways in intrahepatic CCA [10, 11] and identified a novel sphingosine-1-phosphate (S1P) pathway and a potential drug target, sphingosine kinase 1 (SPHK1). Bioactive S1P is now recognized as a critical regulator of cell survival and proliferation, in contrast to ceramide and sphingosine, which induce cell apoptosis and cell growth arrest [12, 13]. Sphingosine kinases (SPHKs) convert sphingosine to S1P and are critical regulators that determine cell fate. Two isoforms of SPHK enzymes with distinct functions, SPHK1 and SPHK2, have been discovered [14, 15]. SPHK1 has oncogenic roles in proliferation, angiogenesis, and transformation [12]. Increased SPHK1 expression has also been observed in gastric cancer [16], breast cancer [17], lung cancer [18], brain tumors [19], colon cancer [20], and lymphoma [21]. High levels of SPHK1 protein are associated with poorer outcomes [13]. However, the role and therapeutic implications of SPHK1 in CCA have not been explored.

In this study of 96 patients with intrahepatic CCA treated with hepatectomy, overexpression of SPHK1 was identified as an independent marker of poor prognosis. We also determined that SPHK1 targeting induced apoptosis in CCA cells *in vitro* and *in vivo*. This preclinical study provides a rationale for clinical trials with SPHK1 in patients with CCA.

## RESULTS

### Analyzing gene signatures of CCA and identification of SPHK1 as a target gene

#### Results of bioinformatics analysis

**Figure 1:**
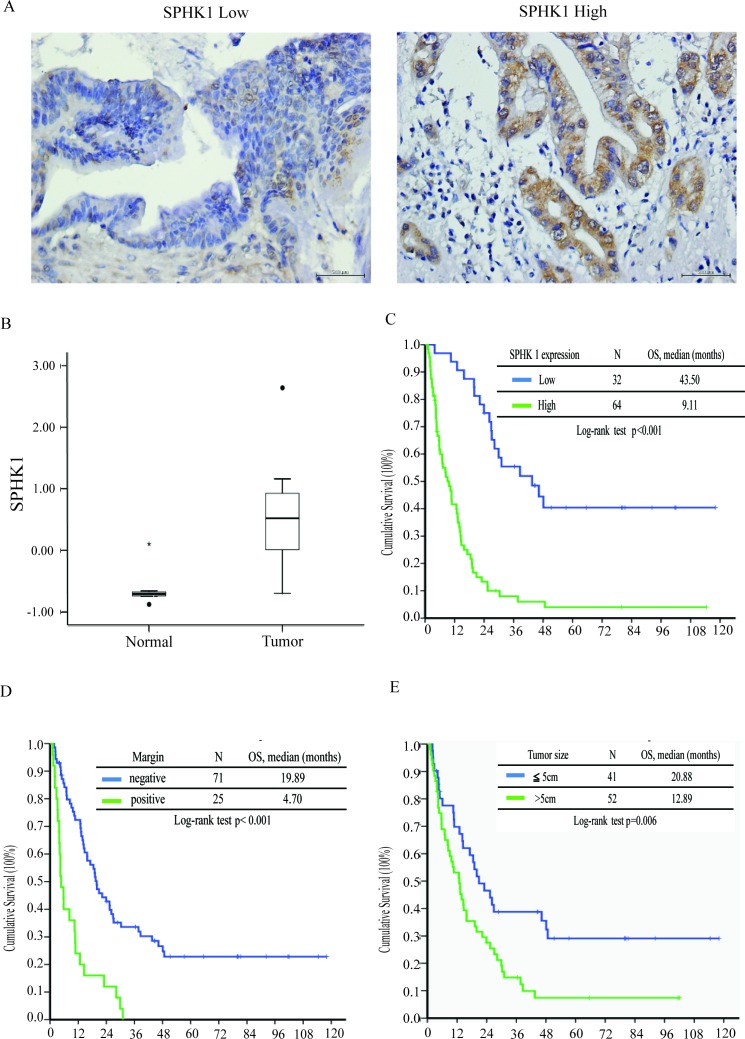
SPHK1 expression, surgical margin, and tumor size correlated with survival in patients with resectable MF-CCA **A.** Immunohistochemical staining of MF-CCA tumors with different staining intensity scores for SPHK1 expression (low and high, respectively). **B.** SPHK1 transcription was higher in CCA samples (*p* < 0.05). **C.** The high-SPHK1 group, **D.** positive surgical margin group, and **E.** large tumor size group showed poorer overall survival (*p* < 0.001).

In total, 320 probes with differential expression, including 49 up-regulated and 271 down-regulated ones, were identified (Figure 1A). Forty-three known genes (Supplement Table 1) corresponding to 49 up-regulated probes were uploaded into PID. Among those genes within the top ten significant pathways (Supplement Table 2), SPHK1 is a known oncogene [12] and has available inhibitors [22]. SPHK1 transcript was significantly over-expressed in tumor tissues (Figure 1B). This gene became the focus of the following studies.

### SPHK1 staining of human MF-CCA specimens and clinicopathological features

Sixty-four of the 96 MF-CCA patient specimens (66%) exhibited strong cytoplasmic immunostaining for SPHK1 (3+). SPHK1 is diffusely expressed in the cytoplasm in MF-CCA (Figure 1A), but is absent in normal cholangiocytes. Various intensities of positive SPHK1 expression could be obtained. Overexpression of SPHK1 was associated with symptoms (*p* = 0.007) and elevated CEA levels (*p* = 0.019); however, only positive symptoms were independently associated with SPHK1 overexpression (Supplementary Table 2).

### Survival and prognostic analysis of MF-CCA patients after hepatectomy

Ninety-six post-hepatectomy patients with MF-CCA (38 men and 58 women; median age 60.9 years, range 22–83 years) were followed up regularly until death. The follow-up duration ranged from 1.4 to 111.8 months (median 14.3 months). Overall survival (OS) rates at 1, 3, and 5 years were 59.5%, 24.4%, and 16.6%, respectively. Univariate log-rank analysis identified the following factors as adverse influences on OS: presence of symptoms, decreased albumin levels, elevated alkaline phosphatase and CEA levels, tumor size >5 cm, positive surgical margin and lymph node status, and SPHK1 immunostaining (Table 2). However, multivariate Cox proportional hazard analysis demonstrated that tumor size >5 cm, non-curative hepatectomy, and positive SPHK1 immunostaining independently predicted an inferior OS rate for MF-CCA patients after hepatectomy (Table 3, Figure 1C, 1D, 1E).

**Table 1:** NCI-Nature Curated pathways obtained by Pathway Interaction Database analysis of up-regulated genes

Pathway Name	Biomolecules in Group	*P* Value
a6b1 and a6b4 Integrin signaling	LAMBS, SFN	1.68E-03*
PDGFR-beta signaling pathway	SFN, SPHK1	1.25E-02*
Alpha6 beta4 integrin-ligand interactions	LAMBS	1.53E-02*
S1P1 pathway	SPHK1	2.89E-02*
p38 signaling mediated by MAPKAP kinases	SFN	2.89E-02*
Sphingosine-l-phosphate (S1P) pathway	SPHK1	2.89E-02*
Signaling events mediated by PRL	PTP4A3	3.15E-02*
Alpha9 beta1 integrin signaling events	ADAMS8	3.42E-02*
Insulin-mediated glucose transport	SFN	3.95E-02*
Class I PI3K signaling events mediated by Akt	SFN	4.86E-02*
Trk receptor signaling mediated by PI3K and PLC-gamma	SFN	4.99E-02*
Signaling mediated by p38-alpha and p38-beta	KRT19	5.12E-02
Beta3 integrin cell surface interactions	SPHK1	5.88E-02
Validated transcriptional targets of deltaNp63 isoforms	SFN	6.38E-02
LKB1 signaling events	SFN	6.38E-02
FoxO family signaling	SFN	6.63E-02
Role of Calcineurin-dependent NFAT signaling in lymphocytes	SFN	7.62E-02
Fc-epsilon receptor I signaling in mast cells	SPHK1	7.98E-02
HIF-1-alpha transcription factor network	PKM	8.71E-02
Beta1 integrin cell surface interactions	LAMBS	9.06E-02
mTOR signaling pathway	SFN	9.06E-02
p73 transcription factor network	SFN	1.01E-01
Regulation of nuclear beta catenin signaling and target gene transcription	SFN	1.03E-01
Glucocorticoid receptor regulatory network	SFN	1.06E-01
ErbB1 downstream signaling	SFN	1.36E-01
Direct p53 effectors	SFN	1.66E-01

* *P* < 0.05

**Table 2:** Univariate analysis of factors influencing the overall survival of patients with MF-CCA

Factors		Survival Time (Months)		*p*
	Events (death)	Median	5-Year (%)	
Gender				0.589
Male (n = 38)	29	18.51	20.9	
Female (n = 58)	47	14.14	13.7	
Age				0.670
≤ (n = 46)	35	14.70	20.4	
>60 (n = 50)	41	14.53	12.3	
Symptoms				0.002*
Negative (n = 16)	8	46.26	43.0	
Positive (n = 80)	68	12.99	11.6	
AST (IU/L)				0.243
≤34 (n= 51)	37	14.40	22.2	
>34 (n = 43)	37	15.85	11.6	
ALT (IU/L)				0.426
≤36 (n = 54)	40	14.53	19.4	
>36 (n = 35)	31	15.85	9.8	
ALP (IU/L)				0.001*
≤94 (n = 36)	23	26.86	27.5	
>94 (n = 55)	50	10.72	9.1	
Bilirubin (total) (mg/dL)				0.392
≤ 1.3(n = 82)	64	15.81	16.8	
>1.3 (n = 14)	12	10.72	14.3	
Albumin (g/dL)				0.023*
≤3.5 (n = 23)	20	5.75	13	
>3.5 (n = 65)	51	19.89	15	
Serum CEA (ng/dL)				0.030*
≤5 (n = 37)	26	19.17	22.6	
>5 (n = 35)	31	12.72	8.9	
Margin				<0.001*
Negative (n = 71)	51	19.89	22.8	
Positive (n = 25)	25	4.70	0	
Size				0.006*
≤5cm (n = 41)	27	20.88	29.1	
>5cm (n = 52)	46	12.89	7.4	
Lymph node				0.016*
Negative (n = 63)	47	20.88	18.4	
Positive (n = 31)	27	12.72	12.9	
Histological differentiation				0.960
Well (n = 3)	2	6.08	33.3	
Moderate (n = 48)	38	15.81	18.6	
Poor (n = 43)	34	14.40	14.6	
Others (n = 2)	2	10.72	0	
SPHK 1 expression				<0.001*
Low (n = 32)	18	43.50	40.4	
High (n = 64)	58	9.11	4.0	
Post-op Chemotherapy				0.383
Without (n = 48)	33	14.40	23.8	
With (n = 48)	43	14.70	10.4	
Post-op Radiotherapy				0.075
Without (n = 84)	64	14.53	19.2	
With (n = 12)	12	7.17	0	

CI: confidence interval; AST: aspartate aminotransferase; ALT: alanine aminotransferase; ALP: alkaline phosphatase; CEA: carcinoembryonic antigen; CA 19-9: carbohydrate antigen 19-9; IU: international unit; op: operation

**Table 3:** Cox's proportional hazards analysis

Factors	Relative Risk (95% Confidence Interval)	P
Symptoms (positive/negative)	—	0.060
ALP (≤94 g/dl/>94 g/dL)	—	0.421
Albumin (≤3.5 g/d1/>3.5g/dI)	—	0.117
Serum CEA (>5 ng/d1/≤5 ng/dL)	—	0.403
Margin (positive/negative)	2.63 (1.40-4.94)	0.003
Tumor size (>5 cm/≤5 cm)	2.29 (1.20-4.38)	0.012
Lymph node (positive/negative)	—	0.688
SPHK 1 expression (high/low)	8.40 (3.58-19.72)	<0.001

ALP: alkaline phosphatase; CEA: carcinoembryonic antigen

### SPHK1 inhibitor, SK1-I, inhibited CCA proliferation *in vitro* and *in vivo*

**Figure 2:**
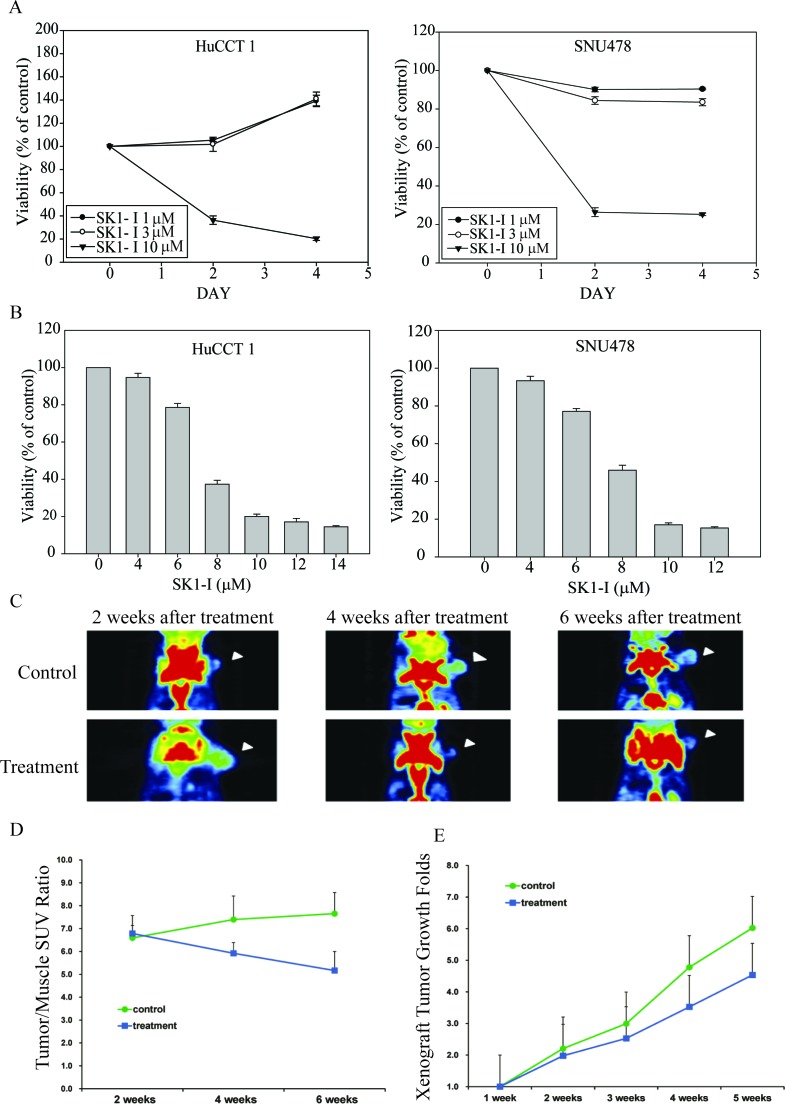
Potent cell growth inhibition induced by SK1-I in CCA cell lines and in the *in vivo* study **A.**, **B.** The antiproliferative effects of SK1-I in the HuCCT1 and SNU478 cell lines were time- and dose-dependent. HuCCT1 and SNU478 cells were exposed to SK1-I (1 μM, 3 μM, and 10 μM) for 48 h and 96 h **A.**. HuCCT1 and SNU478 cells were incubated with various concentrations (4 μM, 6 μM, 8 μM, 10 μM, 12 μM, and 14 μM) of SK1-I for 72 h **B.**. Cell viability was evaluated by MTT assay; data represent the mean ± standard deviation of three independent experiments. **C.** Coronal views of fused CT and PET scans of control and experimental mice revealed the CCA-expressing areas of the xenograft in which the ^18^F-FDG uptake was higher than baseline at 2–6 weeks after the experiment (i.e., weeks 22, 24, and 26). **D.** The tumor-to-muscle ratio of SUV was significantly lower in the experimental groups (10 mg/kg intraperitoneal injection of SK1-I every other day (3 days/week) than in the controls, especially at 4–6 weeks after the experiment (i.e., weeks 24 and 26). **E.** Xenograft tumor growth was significantly higher in the controls than in the experimental groups, especially at 4–5 weeks after the experiment, consistent with the results of the animal PET study (i.e., weeks 24 to 25).

SK1-I exhibited strong time- and dose-dependent antiproliferative effects in HuCCT1 and SNU478 cells (Figure 2A and 2B). CCA xenograft tumors in the control and treated groups were evaluated by animal PET on coronal views. Both groups showed one FDG-avid tumor in the right limb 2 weeks after subcutaneous injection with HuCCT1 (Figure 2C). The SUV ratio of tumor and muscle for both groups is shown in Figure 5. The SK1-I treated group clearly showed a lower T/M ratio at 4 and 6 weeks after treatment when compared with control group (Figure 2D, *p* < 0.0001). Thus, 10 mg/kg intraperitoneal injection of SK1-I every other day (3 days/week) resulted in partial but significant suppression of tumor growth *in vivo* (Figure 2E, *p* < 0.001).

### SK1-I induced apoptosis in CCA cell lines

**Figure 3:**
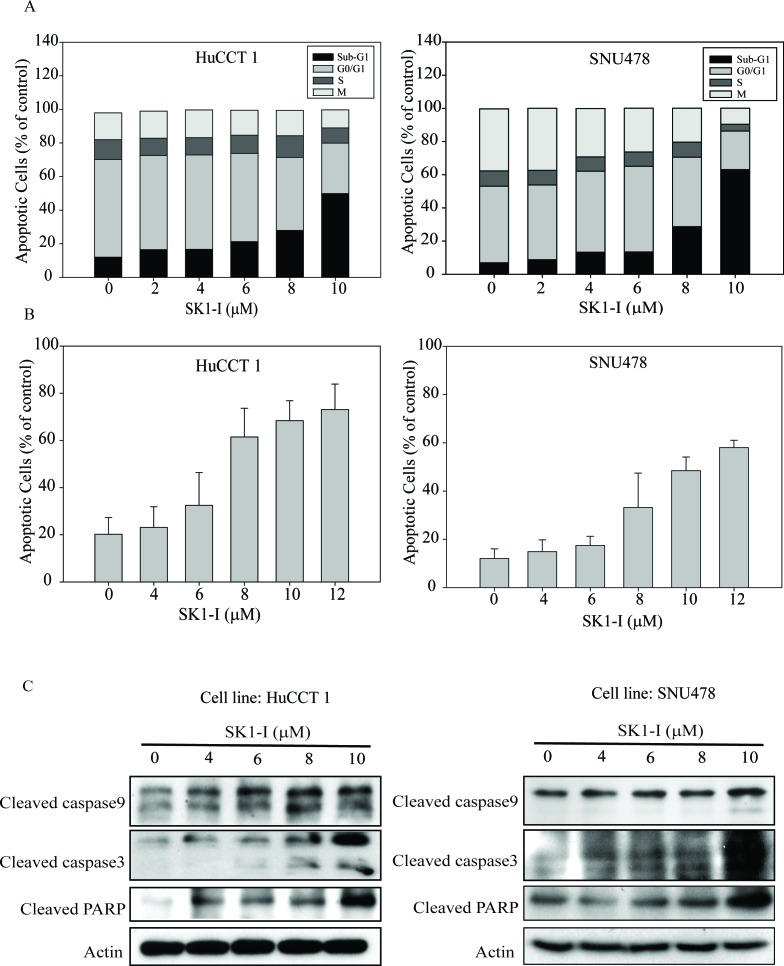
SK1-I induced apoptosis in CCA cell lines **A.** HuCCT1 and SNU478 cells were treated with various concentrations of SK1-I (2 μM, 4 μM, 6 μM, 8 μM, and 10 μM) and harvested at 72 h. The numbers of cells in sub-G1 phase, as determined by flow cytometry, are represented as a percentage of total events. Values represent the mean ± standard deviation of at least three independent experiments. **B.** HuCCT1 and SNU478 cells were treated with various concentrations of SK1-I (4 μM, 6 μM, 8 μM, 10 μM, and 12 μM) for 72 h. Apoptotic cells were measured using the TACS Annexin V-FITC apoptosis detection kit and are represented as a percentage of total events. **C.** Immunoblot analyses of cleaved PARP, caspase 9, and caspase 3 with β-actin loading control.

We investigated the effect of SK1-I on the cell cycle kinetics of HuCCT1 and SNU478 and found that growth arrest by SK1-I was associated with the accumulation of cells in sub-G1 phase (Figure 3A). At 10 μM SK1-I, cell death predominated, with increases in the sub-G1 population to 49.8% and 62.5% in HuCCT1 and SNU478 cells, respectively (Figure 3A). SK1-I also caused a dose-dependent increase in apoptosis within 72 h; 73.1% of HuCCT1 and 61.5% of SNU478 cells were apoptotic after treatment with 12 μM SK1-I (Figure 3B). This was also demonstrated by the dose-dependent increase in cleaved PARP (Figure 3C). PARP cleavage resulted in simultaneous activation of the caspase pathway, as indicated by an increase in levels of cleaved caspases 3 and 9 (Figure 3C).

### SK1-I induced cell apoptosis by increasing intracellular ceramide

**Figure 4:**
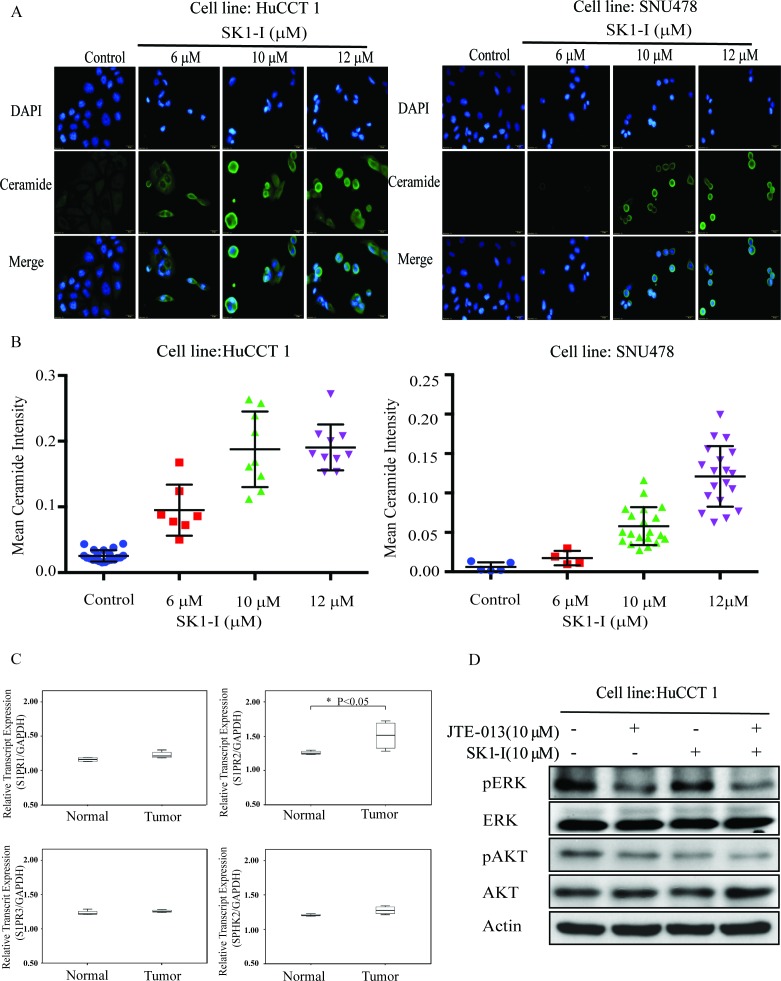
SK1-I increased intracellular ceramide and inhibited ERK and AKT signaling when combined with JTE-013 **A.** HuCCT1 and SNU478 were treated with SK1-I 0 μM, 6 μM, 10 μM, and 12 μM for 48 h. Cells were fixed and stained with anti-ceramide (green) and DAPI (blue) and then imaged by fluorescence microscopy. **B.** The intensity of ceramide expression was greater in HuCCT1 and SNU478 cells treated with SK1-I (6 μM, 10 μM, and 12 μM) than in the controls. **C.** The mRNA expression of S1PR2 was higher in CCA than in normal liver tissue, as determined by qRT-PCR (*p* < 0.05). **D.** Western blot analysis of phosphorylated ERK and AKT after 15 min of treatment with SK1-I 10 μM and JTE-013 10 μM without serum.

According to the proposed ceramide-sphinogosine-S1P rheostat theory, SPHK activity shifts the intracellular balance from the pro-apoptotic ceramide and sphingosine to the mitogenic S1P [13]. Immunofluorescence analysis to measure ceramide expression showed near undetectable levels in control cells and intense expression in HuCCT1 and SNU478 cells treated with SK1-I (6, 10, and 12 μM) (Figure 4A). SK1-I caused a dose-dependent increase the staining intensity of ceramide (Figure 4B). These results suggest that SK1-I regulates interconversion of ceramide-sphinogosine-S1P and directs CCA cell into an apoptotic program.

### Sphingosine 1-phosphate receptor 2 (S1PR2) is the predominant S1P receptor expressed in CCA

We used four paired fresh CCA and normal liver samples to measure transcript levels of SPHK2, S1PR1, S1PR2, and S1PR3 by qRT-PCR. Only S1PR2 was expressed more strongly in tumor tissue than in normal liver tissue (Figure 4C, *p* < 0.05).

### SK1-I combined with JTE-013, an S1P2 antagonist, blocked activation of the ERK/AKT pathways in CCA cell lines

**Figure 5:**
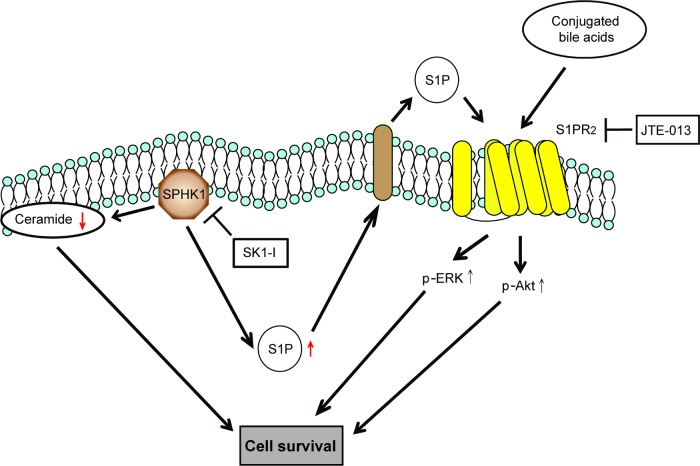
Schematic representation of the effect of SPHK1 and SK1-I on CCA SK1-I modulates the balance of ceramide-S1P and directs the CCA cells into an apoptotic program. SK1-I and JTE-013 synergistically inhibit ERK and AKT signaling by affecting the SPHK1/S1P pathway at multiple nodes.

S1P and conjugated bile acids activate the ERK/AKT pathway through the sphingosine 1-phosphate receptor 2 (S1P2 receptor), and among which S1PR2 is the predominant S1P receptor expressed in human CCA cell lines and tissues [23-25]. We used SK1-I and JTE013 alone and in combination and then used western blotting to assess activation of the ERK/AKT signaling pathways. The use of SK1-I or JTE013 alone did not affect ERK/AKT signaling, but these drugs in combination caused a dramatic reduction in signaling activity (Figure 4D). Thus, simultaneous blockade of SPHK1 and S1PR2 may block the ERK and AKT signaling pathways (Figure 5).

## DISCUSSION

This study demonstrated that SPHK1 is upregulated in intrahepatic CCA tissues in comparison to paired normal liver tissues. Multivariate Cox's proportional hazards model showed that SPHK1 protein expression is an independent unfavorable prognostic indicator of overall survival following hepatectomy. Our study suggests SPHK1 overexpression is common in intrahepatic CCA and represents a novel prognostic marker of disease outcomes.

The role of SPHK1 has been characterized in a rat CCA model [26]. Dumur et al. developed an orthotopic CCA rat model based on bile duct inoculation. They also developed spontaneously transformed low-grade malignant rat BDE1 cholangiocytes (BDEsp cells) and high-grade malignant erbB-2/neu-transformed BDE1 cholangiocytes (BDEneu cells) that closely mimic the clinical features of early and advanced human CCA disease. Microarray analysis showed SPHK1 was overexpressed in both cell types, suggesting SPHK1 is a putative molecular target with possible relevance to progressive human cancer. Our findings supported their hypothesis in the rat model.

The ceramide-sphinogosine-S1P rheostat was the earliest model in the field of S1P research [27]. According to this model, ceramide and sphingosine induce apoptosis, whereas S1P promotes cell growth, proliferation, and survival. SPHK1 is responsible for the conversion of sphingosine to S1P. Therefore, SK1-I, which is an ATP-competitive SPHK1 inhibitor, might modulate this rheostat toward cell death by increasing ceramide. Indeed, in our *in vitro* study, SK1-I induced CCA cell apoptosis, as evidenced by the accumulation of cells in sub-G1 phase and the increasing numbers of Annexin V-stained apoptotic cells, as well as increasing amounts of cleaved caspase 3, caspase 9, and PARP. We also found that intracellular ceramide concentrations increased after SK1-I treatment. Thus, SK1-I modulates the balance of ceramide-sphingosine-S1P and directed the CCA cells into an apoptotic program.

Understanding the mechanisms leading to CCA formation is important for the development of new and effective therapeutic options to treat this devastating disease. Bile acids may play a role in CCA development and exhibit pro-proliferative effects by activating ERK1/2 and AKT signaling pathways in murine and human hepatocytes [25]. Studer et al. showed that conjugated bile acids activate ERK1/2 and AKT signaling through sphingosine-1-phosphate receptor 2 (S1PR2) in rodent hepatocytes [28]. Liu et al. reported that conjugated bile acids also sustain CCA growth and invasiveness through activation of S1PR2 [24] and demonstrated that S1PR2 is the predominant S1P receptor expressed in human CCA cell lines. S1PR2 expression is also higher in human CCA tissues compared to non-tumor samples (Figure 4C). We found diffuse SPHK1 expression in the cytoplasm of human CCA, but the protein was absent in normal cholangiocytes. S1P and conjugated bile acids activated ERK1/2 and AKT signaling through S1PR2 in CCA. We also identified synergy between an SPHK1 inhibitor (SK1-I) and a S1PR2 selective antagonist (JTE-013) in the blockade of ERK1/2 and AKT signaling. Simultaneous blockade of SPHK1 and S1PR2 may also block CCA progression.

In conclusion, SPHK1 overexpression was identified as an independent poor prognostic factor, suggesting the SPHK1/S1P pathway is a potential therapeutic target in CCA. The SPHK1 inhibitor SK1-I had potent antitumor activity against CCA *in vivo* and *in vitro*. These preclinical findings provide the rationale for clinical trials of this SPHK1 inhibitor in CCA patients.

## PATIENTS AND METHODS

### Bioinformatics analysis

Normalized expression profiles were obtained on the NimbleGen Platform for seven paired intrahepatic CCA and adjacent normal liver tissues. Gene expression data were analyzed by dChip [29, 30]. Differences in expression between tumor and normal liver tissues were compared by using the *t*-test [31, 32]. To identify important tumor targets, the list of upregulated genes was uploaded to the Pathway Interaction Database (PID, http://pid.nci.nih.gov/) [33]. A PID pathway is a network of five event types (gene regulation, molecule transport, small-molecule conversion, protein–protein interactions and black-box processes whose internal composition is not provided) connected by four participant molecules (small molecules, RNA, proteins, and complexes). NCI-Nature Curated pathways, curated by the Nature Publishing Group editors based on potential drug targets, suggestions made by users and reviewers, and other molecules known to be of interest to the cell signaling community, were exported for further analysis.

### Clinicopathological features of patients with mass forming-CCA (MF-CCA)

From the archives of Chang Gung Memorial Hospital, 96 MF-CCA patients who had undergone hepatectomy between 1989 and 2006 were selected based on the availability of sufficient tumor samples. Intrahepatic CCA was defined as carcinoma that arose from distal second order (or higher) branches of the intrahepatic ducts. Curative resection was defined as a negative resection margin observed during histopathological examination. Surgical mortality was defined as death that occurred within 1 month of surgery. Laboratory tests were conducted on the day before surgery. Serum carbohydrate antigen 19-9 (CA 19-9) and carcinoembryonic antigen (CEA) were measured by radioimmunoassay. The tumors were preoperatively evaluated by abdominal ultrasonography (US), endoscopic retrograde cholangiopancreatography, percutaneous transhepatic cholangiography, computed tomography (CT), magnetic resonance cholangiopancreatography (MRCP), and hepatic arteriography, as appropriate. Tumor stage was defined according to the pathological tumor node metastasis classification proposed by the American Joint Committee on Cancer, 6^th^ edition. This retrospective study was approved by the institutional review board at Chang Gung Memorial Hospital (clinical study No. 99-2886B and 102-5813B). All patients provided informed consent before taking part in the immunostaining study.

### Immunohistochemical staining of SPHK1 in human CCA

Hematoxylin and eosin-stained slides from each case were reviewed. Specimens from MF-CCA patients who had undergone hepatectomy were fixed in formalin and embedded in paraffin. A 4-μm section of each specimen was stained for stratifin. The primary antibody against SPHK1 (rabbit anti-SPHK1 antibody, ab61148, Abcam, Cambridge, UK) was diluted (1:200) and added to the slides that were then incubated overnight at 4°C. The slides were washed three times for 5 min in TBST before visualization with the DAKO LSAB2 System, Peroxidase (DAKO A/S, No K0675). Control slides were incubated with a secondary antibody alone. After washing three times in TBST for 5 min each, the slides were mounted. Immunohistochemical staining of stained target cells was evaluated in 10 optical microscope fields per tissue section (400× magnification), and the average staining percentage was calculated. Staining intensity was scored as 1 (mild), 2 (moderate), or 3 (intense). H-scores were calculated as the percentage of positive staining (0–100) × the correspondent staining intensity (0–3). Specimens with H-scores of <50 and ≥50 were classified as immunohistochemically low and immunohistochemically high expression, respectively [34].

### Follow-up study

Follow-up evaluation included physical examinations and blood chemistry tests at each visit. Additionally, serum levels of CEA and CA 19-9 were measured, and the remnant liver was examined by US every 3 months. When a new lesion was detected by US or elevated levels of CEA/CA 19-9 were noted, abdominal CT or MRCP was performed for confirmation. When patients complained of bone pain, bone scans were performed to detect metastasis. If any of the abovementioned procedures indicated recurrence, the patient was readmitted for a more comprehensive assessment, including angiographic evaluation or magnetic resonance imaging (MRI). The methods for treating recurrence included surgery, systemic chemotherapy, external beam radiotherapy, intraluminal radiotherapy, interventional radiological therapy, and conservative treatment.

### Cell lines

Two intrahepatic CCA cell lines, HuCCT1 and SNU478, were obtained from the Japanese Collection of Research Bioresources (Osaka, Japan) and the Korean Cell Line Bank (Seoul, Korea), respectively [35, 36] (35, 36). HuCCT1 and SNU478 cells were routinely cultured in RPMI 1640 and Dulbecco's modified Eagle's medium (Gibco, Grand Island, NY), respectively, supplemented with 10% heat-inactivated fetal bovine serum, 100 μg/mL streptomycin, and 100 μg/mL penicillin, in a humidified atmosphere containing 5% CO_2_ at 37°C.

### Reagents

SK1-I was purchased from (Enzo Life Sciences, NY). JTE-013 was purchased from Cayman Chemical (Ann Arbor, MI). For the *in vitro* experiments, 10 mM stock solutions of SK1-I and JTE-013 were prepared in 100% DMSO and stored at −20°C. For administration, optimized aqueous SK1-I salts were formulated in D5W. SK1-I was delivered by intraperitoneal (i.p.) injection at a dose of 10 mg/kg.

### Viability assay

Cell viability was determined using the TACS tetrazolium salt 3-(4, 5-dimethylthiazol-2-yl)-2,5-diphenyltetrazolium bromide (MTT) cell proliferation assay kit (Trevigen, Gaithersburg, MD), according to manufacturer instructions. MTT is used to determine cell viability in cell proliferation and cytotoxicity assays. Briefly, cells were seeded at a concentration of 1500 cells per well (HuCCT1 cells and SNU478 cells) in 100 μL culture medium in 96-well microplates. At 24 h post-seeding, the cells were treated with DMSO or SK1-I for 48, 72, and 96 h; the cells were then incubated in medium containing MTT for 2 h. The optical density at 570 nm was measured using a microplate reader (Spectral Max250; Molecular Devices, Sunnyvale, CA).

### Cell cycle analysis

Cells were plated in 6-well plates, incubated for 24 h, and then treated with DMSO and SK1-I. The cells were trypsinized and fixed in 70% ethanol at −20°C, washed, and incubated with 10 mg/mL RNase A (Sigma) for 15 min at 37°C, and then stained with 200 μg/mL propidium iodide (Sigma) for 1 h at room temperature before analysis on a FACSCalibur machine (Becton Dickinson, Franklin Lakes, NJ). CellQuest software (Becton Dickinson) was used to model the cell cycle distribution. Experiments were performed in triplicate and data are expressed as the mean ± standard deviation.

### Apoptotic cell death

Apoptosis was measured using the FITC Annexin V apoptosis detection kit (BD Biosciences), according to manufacturer instructions. After a 24-h incubation, cells were treated with DMSO, SK1-I for 72 h. Cells were collected and stained with Annexin V and propidium iodide (PI), and then analyzed using a FACSCalibur machine. The data were analyzed using CellQuest software (BD). Experiments were performed in triplicate, and data are expressed as mean ± standard deviation.

### Western blotting

Whole cell lysates of CCA cell lines were obtained in Pierce RIPA buffer (Thermo Scientific, Rockford, IL). Protein samples were separated on 8%–12% gradient sodium dodecyl sulfate-polyacrylamide gels (SDS-PAGE) and transferred to Immobilon-P (Millipore, Bedford, MA) membranes. Antigen-antibody complexes were detected using the ECL blotting analysis system (Millipore). Primary antibodies against the following targets were used: cleaved poly (ADP-ribose) polymerase (c-PARP, 9541S; Cell Signaling), β-actin (Abcam ab6276), caspase 9 (9508; Cell Signaling), caspase 3 (9662; Cell Signaling), SPHK1 (9252; Cell Signaling), phospho-Akt (4058; Cell Signaling), AKT (9272; Cell Signaling), phospho-p42/44 MAPK (Erk1/2) (4376; Cell Signaling), and ERK (SC-135900; Santa Cruz).

### Immunofluorescence microscopy

HuCCT1 and SNU478 cells were grown on four-chamber Permanox slides for 24 h and then treated with SK1-I (0, 6, 10, and 12 μM) for 48 h. Cells were fixed in 4% paraformaldehyde for 10 min at room temperature and then blocked with 5% BSA in TBS for 1 h at room temperature. Cells were incubated overnight at 4°C with ceramide (1:10; Enzo Life Sciences, NY), followed by incubation with FITC-conjugated goat anti-rabbit IgG (Bios, Beijing, China) for 60 min at room temperature. Explants and cells were washed in TBS and then incubated in TBS buffer + DAPI for 10 min prior to analysis by fluorescence microscopy. The expression of ceramide in the image was then analyzed by CellProfiler [37], which automatically calculated the staining intensity of ceramide per cell.

### Quantitative reverse transcription-polymerase chain reaction (qRT-PCR)

Reverse transcription was performed using 3 μg total RNA with a reverse transcriptase by using a cDNA kit (Invitrogen), and real-time PCR and quantitative PCR were performed to assess expression of S1PR1, S1PR2, S1PR3, and SPHK2 by using primers designed for the human mRNA sequences [38-40]. Cycling conditions for real-time PCR included an initial denaturation cycle at 95°C for 15 min, followed by 40 amplification cycles of 95°C for 15 sec, 60°C for 60 sec, and a final extension at 72°C for 30 sec, followed by melt curve analysis.

### Tumor xenograft establishment

Twelve male BALB/c nude mice (age, 4 weeks) were used in the experiments. The animals were divided into control (n = 6) and experimental groups (n = 6) and housed in a specific pathogen-free animal facility with a 12/12-h light/dark cycle (light from 8:00 AM to 8:00 PM) at an ambient temperature of 20°C. HuCCT1 xenograft was established by subcutaneous injection of 10^6^ tumor cells. SK1-I was prepared in PBS (1 mg/mL) and administered at a dose of 10 mg/kg via an intraperitoneal injection every other day (3 days/week) over a 6-week period. The control group received PBS. All animals were treated under anesthesia, and all efforts were made to minimize suffering.

### Treatment efficacy evaluation by positron emission tomography (PET)

All mice underwent 18F-fluorodeoxyglucose (^18^F-FDG) PET studies at the molecular imaging center, Chang Gung Memorial Hospital, Linkou. Serial PET scans were performed before treatment and at weeks 2, 4, and 6 after SK1-I treatment by using the Inveon^TM^ system (Siemens Medical Solutions, Malvern, PA, USA). Details regarding radioligand preparation, scanning protocols, and optimal scanning time are described in our previous report [41]. Quantification of ^18^F-FDG uptake in the xenograft and surrounding normal muscle was performed according to the recommendations of the European Organization for Research and Treatment of Cancer [42] by calculating the standardized uptake value (SUV) using the following formula:

Decay corrected tissue activity (Bq = mL)

SUV∼

Injected dose (Bq) = Body weight (g)

The xenograft regions of interest (ROI) were determined according to the largest diameter of the selected xenograft in transverse images, and the ROIs of surrounding normal muscle were determined from the same transverse images. The xenograft and muscle mean SUVs (SUV_mean_) and the xenograft-to-muscle (X/M) radioactivity ratio were calculated for comparison.

### Statistical analysis

All data are presented as means and standard deviations (SD). Differences between experimental animals and controls were calculated using the Mann-Whitney *U* test or the Kruskal-Wallis test. The SUV ratio between experimental animals and controls was calculated by using the nonlinear trend test. The overall survival rates were calculated with the Kaplan-Meier method. Sixteen clinicopathological variables were selected for survival difference analysis by the log-rank test (univariate). The Cox proportional hazards model was employed for multivariate regression analysis. The statistical software SPSS for Windows (SPSS version 13.0, Chicago, IL) was used for the statistical analysis and *P* ≤ 0.05 was considered statistically significant.

## SUPPLEMENTARY MATERIAL TABLES AND FIGURE



## Abbreviations

SPHK1: sphingosine kinase 1
S1P: sphingosine-1-phosphate
S1PR2: sphingosine-1-phosphate receptor 2
MF-CCA: mass forming cholangiocarcinoma
PARP: Poly ADP ribose polymerase
