# Supplementary material for: Identification of SPHK1 as a therapeutic target and marker of poor prognosis in cholangiocarcinoma
Source: Oncotarget. 2015 Jun 2;6(27):23594–608. doi: 10.18632/oncotarget.4335 (PMC4695139; doi:10.18632/oncotarget.4335)
Supplement: Supplementary file 1 [file oncotarget-06-23594-s001.pdf]

Identification of SPHK1 as a therapeutic target and marker of poor prognosis in cholangiocarcinoma

Supplementary Material

Supplementary Table 1. Up-regulated probe IDs and corresponding genes

Probes	Genes	Fold change
NM_032432	ABLIM2	2.13
NM_001109	ADAM8	2.31
NM_198576	AGRN	2.04
NM_001012302	ANO9	2.01
NM_033520	C19orf33	6.87
NM_014008	CCDC22	2.24
NM_016564	CEND1	2.56
NM_000095	COMP	3.44
NM_001311	CRIP1	2.8
NM_004395	DBN1	2.93
NM_033178	DUX4L1	2.22
XM_374852	DUX4L11	2.12
XM_496731	DUX4L4	2.25
NM_001023569	DUX4L9	2.05
NM_000149	FUT3	2.03
NM_053044	HTRA3	2.2
NM_002276	KRT19	5.73
NM_005556	KRT7	5.18
NM_000228	LAMB3	5.25
NM_002304	LFNG	2.28
XM_927367	LOC377711	2.51
NM_001013637	LOC388022	2.15
XM_372046	LOC389667	2.55
XM_926221	LOC653091	2.2
XM_927996	LOC653541	2.19
NM_173481	MISP	3.12
NM_005940	MMP11	3.37
NM_015392	NPDC1	2.03
NM_022463	NXN	2.51
NM_005764	PDZK1IP1	3.51
NM_182470	PKM	4.06

NM_002654	PKM	3.98
NM_017514	PLXNA3	2.03
NM_002773	PRSS8	2.11
NM_007079	PTP4A3	2.05
NM_020387	RAB25	2.38
NM_014624	S100A6	2.57
NM_006142	SFN	4.66
NM_004207	SLC16A3	5.01
NM_198857	SLC6A10P	2.63
NM_021972	SPHK1	2.75
XM_925801	TBC1D3D	2.03
NM_017899	TESC	9.29
NM_024339	THOC6	2.23
NM_213674	TPM2	3.23
NM_003294	TPSAB1	2.29
NM_024164	TPSB2	2.57
NM_005709	USH1C	3.71
NM_080734	WFDC2	2.12

Supplementary Table 2. Clinicopathological features and SPHK1 expression in patients with cholangiocarcinoma

	SPHK1 low expression (n = 32)	SPHK1 high expression (n = 64)	*p*
Age (years)	62.04 ± 12.21	59.41 ± 11.57	0.306
Gender			0.883
Male	13 (40.6%)	25 (39.1%)	
Female	19 (59.4%)	39 (60.9%)	
Symptom			0.007*
No	10 (31.3%)	6 (9.4%)	
Yes	22 (68.8%)	58 (90.6%)	
AST (IU/L)			0.780
≤34	18 (56.3%)	33 (53.2%)	
>34	14 (43.8%)	29 (46.8%)	
ALT (U/L)			0.851
≤36	18 (62.1%)	36 (60.0%)	
>36	11 (37.9%)	24 (40.0%)	
ALP (U/L)			0.245
≤94	14 (48.3%)	22 (35.5%)	
>94	15 (51.7%)	40 (64.5%)	
Bilirubin (total) [illegible]			0.131
≤1.3	30 (93.8%)	52 (81.3%)	
>1.3	2 (6.3%)	12 (18.8%)	
Albumin (g/dl)			0.065
≤3.5	4 (13.8%)	19 (32.2%)	
>3.5	25 (86.2%)	40 (67.8%)	
Serum CEA (ng/mL)			0.029*
≤5	15 (71.4%)	22 (43.1%)	
>5	6 (28.6%)	29 (56.9%)	
Size (cm)			0.405
≤5	16 (50.0%)	25 (41.0%)	
>5	16 (50.0%)	36 (59.0%)	
Lymph node			0.258
Negative	24 (75.0%)	39 (60.9%)	
Positive	8 (25.0%)	23 (35.9%)	
Differentiated			0.726
Well	1 (3.1%)	2 (3.1%)	
Moderate	18 (56.3%)	30 (46.9%)	
Poorly	12 (37.5%)	31 (48.4%)	
Other	1 (3.1%)	1 (1.6%)	
Margin			0.100
Negative	27 (84.4%)	44 (68.8%)	
Positive	5 (15.6%)	20 (31.3%)	
Post Chemotherapy			0.386
Without (n = 48)	18 (56.3%)	30 (46.9%)	
With (n = 48)	14 (43.8%)	34 (53.1%)	
Post Radiotherapy			0.745

Without (n = 84)	29 (90.6%)	55 (85.9%)	
With (n = 12)	3 (9.4%)	9 (14.1%)	

*statistically significant by multilogistic regression analysis: $p = 0.006$ for symptom

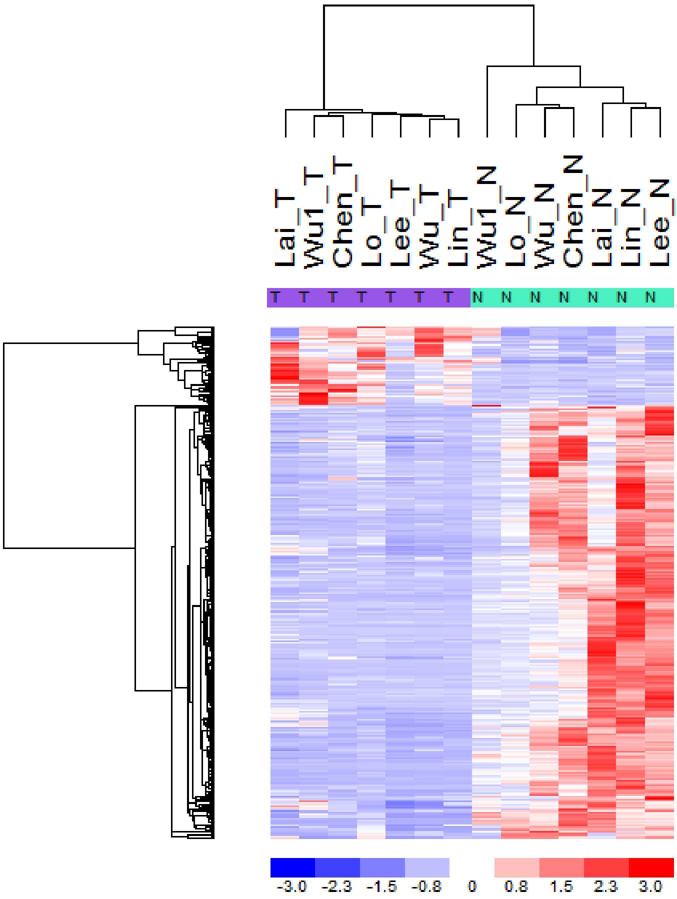

Supplementary Figure 1.

Gene expression heat map of seven paired patients with intrahepatic cholangiocarcinoma
